# Supplementary material for: BRCT domains of the DNA damage checkpoint proteins TOPBP1/Rad4 display distinct specificities for phosphopeptide ligands
Source: eLife. 2018 Oct 8;7:e39979. doi: 10.7554/eLife.39979 (PMC6175577; doi:10.7554/eLife.39979)
Supplement: Supplementary file 1. [file elife-39979-supp1.docx]

**SUPPLEMENTARY FILE 1**

|  | **GgTOPBP1-BRCT0,1,2**  **HsRAD9-pS387** | **SpRad4-BRCT1,2**  **SpSld3-pT636,pT650** | **SpRad4-BRCT1,2**  **SpMdb1-pT113** |
| --- | --- | --- | --- |
| **Wavelength** | 0.91407 | 0.9795 | 0.97950 |
| **Resolution range** | 38.7 - 2.33 (2.413 - 2.33) | 33.22 - 1.773 (1.836 - 1.773) | 31.94 - 1.77 (1.833 - 1.77) |
| **Space group** | P 1 21 1 | P 21 21 21 | P 1 21 1 |
| **Unit cell** | 59.05 34.07 66.67 90 103.57 90 | 39.4 59.17 120.43  90.00 90.00 90.00 | 50.17 40 54.96  90.00 105.16 90.00 |
| **Total reflections** | 33448 (3411) | 80896 (8170) | 50974 (2487) |
| **Unique reflections** | 11242 (1122) | 27527 (2735) | 19339 (1453) |
| **Multiplicity** | 3.0 (3.0) | 2.9 (3.0) | 2.6 (1.7) |
| **Completeness (%)** | 98.97 (99.12) | 97.90 (99.45) | 93.07 (69.24) |
| **Mean I/sigma(I)** | 9.72 (2.12) | 15.69 (2.08) | 16.52 (2.08) |
| **Wilson B-factor** | 39.53 | 26.18 | 20.48 |
| **R-merge** | 0.08416 (0.529) | 0.04109 (0.5432) | 0.04735 (0.4284) |
| **R-meas** | 0.1024 (0.6413) | 0.04996 (0.6616) | 0.05807 (0.5743) |
| **Rpim** | 0.0575 (0.3581) | 0.02795 (0.3711) | 0.03309 (0.3778) |
| **CC1/2** | 0.995 (0.658) | 0.02795 (0.3711) | 0.998 (0.698) |
| **CC*** | 0.999 (0.891) | 1 (0.942) | 1 (0.907) |
| **Reflections used in refinement** | 11241 (1122) | 27524 (2735) | 19338 (1452) |
| **Reflections used for R-free** | 1124 (112) | 1385 (129) | 991 (89) |
| **R-work** | 0.2012 (0.2547) | 0.1917 (0.3017) | 0.1664 (0.2730) |
| **R-free** | 0.2405 (0.3092) | 0.2278 (0.3298) | 0.2116 (0.3625) |
| **CC(work)** | 0.939 (0.796) | 0.959 (0.867) | 0.963 (0.850) |
| **CC(free)** | 0.913 (0.724) | 0.958 (0.852) | 0.927 (0.715) |
| **Number of non-hydrogen atoms** | 2115 | 1907 | 1845 |
| **Protein residues** | 259 | 208 | 191 |
| **RMS(bonds)** | 0.002 | 0.007 | 0.007 |
| **RMS(angles)** | 0.52 | 0.91 | 0.83 |
| **Ramachandran favored (%)** | 96.37 | 99.49 | 99.45 |
| **Ramachandran allowed (%)** | 3.63 | 0.00 | 0.55 |
| **Ramachandran outliers (%)** | 0 | 0.51 | 0.00 |
| **Rotamer outliers (%)** | 2.15 | 0.55 | 0.00 |
| **Clashscore** | 1.96 | 5.06 | 2.90 |
| **Average B-factor** | 46.63 | 34.55 | 22.63 |

Statistics for the highest-resolution shell are shown in parentheses.
